# Transcriptomics Analysis Reveals the Immune Response Mechanism of Rabbits with Diarrhea Fed an Antibiotic-Free Diet

**DOI:** 10.3390/ani11102994

**Published:** 2021-10-19

**Authors:** Li Chen, Kun Du, Xue Bai, Jiahao Shao, Tao Tang, Siqi Xia, Huimei Fan, Jie Wang, Xianbo Jia, Songjia Lai

**Affiliations:** 1 College of Animal Science and Technology, Sichuan Agricultural University, Chengdu 611130, China; chenl2020302120@163.com (L.C.); Dukun1672@163.com (K.D.); 2 Department of Animal Science, University of Florida, Gainesville, FL 32611, USA; baixue333work@163.com (X.B.); shaojh1997@163.com (J.S.); m18483220592@163.com (T.T.); xiasiqi2020@163.com (S.X.); fanhuimei1998@163.com (H.F.); 3 Farm Animal Genetic Resources Exploration and Innovation Key Laboratory of Sichuan Province, College of Animal Science and Technology, Sichuan Agricultural University, Chengdu 611130, China; wjie68@163.com (J.W.); jaxb369@sicau.edu.cn (X.J.)

**Keywords:** transcriptomic analysis, no-antibiotic diet, diarrhea, rabbit

## Abstract

**Simple Summary:**

Feeding an antibiotic-free diet is an inevitable trend in animal husbandry in China. In this study, high-throughput sequencing was used to analyze the gene expression differences in various intestinal segments of rabbits. Our analysis identified 168, 593, 2069, 334, 321, and 1423 DEGs in the comparison groups S_Z (the duodenum of healthy rabbits) vs. S_B (diarrhea in the duodenum of rabbits), K_Z (healthy rabbit jejunum) vs. K_B (rabbits with diarrhea in the jejunum), H_Z (healthy rabbit ileum) vs. H_B (rabbits with diarrhea in the ileum), M_Z (healthy cecum of rabbits) vs. M_B (rabbit with diarrhea in the cecum), J_Z (healthy rabbit colon) vs. J_B (colon of rabbits with diarrhea), and Z_Z (healthy rabbit rectum) vs. Z_B (rectum of rabbits with diarrhea), respectively. The reproducibility and repeatability of the results were validated by RT-qPCR. Enrichment analyses of GO annotations and KEGG pathways revealed the host DEGs that are potentially related to acute inflammation, stress response, tissue dehydration, adaptive immune response, protein binding, activation of related enzymes, migration of immune cells, and so on. In this descriptive study, our findings revealed the changes in the host transcriptome expression profile while feeding an antibiotic-free diet and suggested that feeding an antibiotic-free diet alters the host’s metabolic network and the expression of antiviral proteins.

**Abstract:**

China officially promulgated the announcement of banning the use of antibiotics in the animal industry in 2020. However, the prohibition of antibiotics in the animal industry would reduce the feed conversion rate and increase the mortality of animals. In order to obtain information about the pathogenesis and host immune response of rabbits with diarrhea after being fed an antibiotic-free diet, we first analyzed the intestinal tissue sections of rabbits. Secondly, the gene expression differences of rabbit intestinal segments were analyzed by high-throughput sequencing. Our analysis identified 168, 593, 2069, 334, 321, and 1423 DEGs in the comparison groups S_Z (the duodenum of healthy rabbits) vs. S_B (diarrhea in the duodenum of rabbits), K_Z (healthy rabbit jejunum) vs. K_B (rabbits with diarrhea in the jejunum), H_Z (healthy rabbit ileum) vs. H_B (rabbits with diarrhea in the ileum), M_Z (healthy cecum of rabbits) vs. M_B (rabbits with diarrhea in the cecum), J_Z (healthy rabbit colon) vs. J_B (colon of rabbits with diarrhea), and Z_Z (healthy rabbit rectum) vs. Z_B (rectum of rabbits with diarrhea), respectively. The reproducibility and repeatability of the results were validated by RT-qPCR. Enrichment analyses of GO annotations and KEGG pathways revealed the host DEGs that are potentially related to acute inflammation, stress response, tissue dehydration, adaptive immune response, protein binding, activation of related enzymes, migration of immune cells, and so on. In this descriptive study, our findings revealed the changes in the host transcriptome expression profile after feeding an antibiotic-free diet and suggested that feeding an antibiotic-free diet alters the host’s metabolic network and the expression of antiviral proteins, which provides a theoretical basis for further study on the immune response of animals fed an antibiotic-free diet.

## 1. Introduction

Since the first use of antibiotics in piglets and chickens in 1949, it has been found that feeding diets containing antibiotics could maintain animal intestinal health and reduce livestock mortality [1,2]. However, feeding an antibiotic diet would gradually weaken the immune ability of animals, causing animal resistance to antibiotics and forming a vicious circle, and leading to human health problems caused by antibiotic residues in meat [3]. Therefore, the animal industry and the consumer market were increasingly calling for the abolition of the use of feed antibiotics [4,5]. For example, Sweden, the European Union, and China banned the use of antibiotics in animal feed in 1986, 2006, and 2020, respectively.

After the animal breeding industry banned the use of antibiotics, it caused a series of economic losses, such as increased animal mortality, increased breeding costs, and decreased quality of animal products [6,7]. In particular, some bacterial diseases have reappeared and spread, affecting animal health and welfare [8,9,10,11]. For animals, the intestine is an important organ for nutrient digestion and absorption, microbial defense, immune response, and hormone secretion [12,13,14,15,16]. The integrity of the intestinal mucosal barrier determines the health of the intestine [17]. According to reports, the ban on antibiotics in the EU would cause epidemic necrotizing enteritis in livestock and poultry [18]. Digestive system diseases account for 70% of all diseases in rabbits currently [19]. The incidence rate of diarrhea is the highest among all digestive system diseases in rabbits, especially weaning ones [20]. Diarrhea could lead to low immunity and increased mortality of rabbits, which restricts the development of the rabbit industry [21].

In this study, rabbits were fed an antibiotic-free diet, and a large number of rabbits had diarrhea. However, the immune mechanism of diarrhea in rabbits was not clear. Nowadays, high-throughput transcriptome technology can conduct very sensitive experiments in a cost-effective way. In this study, the differential genes of the rabbit intestinal transcriptome were analyzed by high-throughput technology so as to explore the pathogenesis of diarrhea in rabbits fed an antibiotic-free diet in order to provide a theoretical basis for the development of animal intestinal disease treatment.

## 2. Materials and Methods

### 2.1. Ethics Statement

Experimental procedures in this study were approved by the Institutional Animal Care and Use Committee of the College of Animal Science and Technology, Sichuan Agricultural University, Sichuan, 611130, China.

### 2.2. Animals

Two hundred Hyplus female rabbits at 35 days of age were selected from the Zhongtian rabbit farm (Township, Leibo, Liangshan Yi Autonomous Prefecture, Sichuan, China) in three farm districts. Ten days before weaning, they were fed an antibiotic-containing diet together with female rabbits, and for 10 days after weaning, they were freely fed an antibiotic-free diet. All rabbits were raised under standard farm conditions and received routine vaccinations. At the end of the experiment, six rabbits were selected from the group in a healthy state and treated as the normal group (CON), and six rabbits were selected from the diseased rabbits according to the disease criteria and treated as the diseased group (DIA). The selection of the DIA standard was based on phenotypic differences, weight differences, stool differences, urine differences, and intestinal histopathological diagnosis.

Feed ingredients and additives were prepared according to the nutritional requirements of INRA in France. Each rabbit was placed in a clean cage (60 × 60 × 50 cm) and placed in an environmentally controlled room (21–23 ℃, 60–75% humidity, 14 h of light (60 LX)) [22].

### 2.3. Collection of Rabbit Intestinal Tissue Samples

After fasting for 24 h, rabbits were blood let with electroshock. After that, we collected duodenum, jejunum, ileum, cecum, colon, and rectum intestinal tissue samples. Then the contents of the intestines of the rabbits were washed with normal saline. Samples placed in 3 mL cryotubes were stored in liquid nitrogen at −80 ℃. Finally, the collected intestinal tissue samples were used for transcription analysis.

### 2.4. Morphological Section Analysis of Rabbit Intestine

We selected seven rabbits with diarrhea and three healthy rabbits for intestinal morphological section analysis. The intestinal tissue specimens were fixed with 4% paraformaldehyde, trimmed, dehydrated, embedded in paraffin, sectioned, dewaxed with xylene, stained with hematoxylin and eosin (HE), dehydrated with alcohol, and sealed with resin. The histological changes of the whole tissue sections were observed under a microscope (HE, bar = 100 μm, 100×), and normal areas and obvious lesion areas were recorded with a microscope imaging system.

### 2.5. RNA Extraction, cDNA Library Construction, and Sequencing

The duodenum, jejunum, ileum, cecum, colon, and rectum of the DIA and CON were selected for RNA-seq analysis (n = 3). Total RNA was extracted from intestinal tissues of rabbits with diarrhea and healthy control rabbits by standard extraction method. The effective RNA was screened by Agilent 2100 biological analyzer (Agilent RNA 6000 nano). The screening criteria were RNA 7.0 Rin value and 28s/18S > 1.8. The purified double-stranded cDNA was repaired at the end, an A tail was added, and the sequencing connector was connected. The 370–420 bp cDNA was screened, amplified by PCR, and purified again, and the library was finally obtained. The NEBNext^®^ Ultra™ RNA Library Prep Kit for Illumina^®^ (San Diego, CA, USA) [23] was used to construct the library. Then Illumina sequencing was performed.

### 2.6. RNA-Seq Data Analyses

Image data measured by high-throughput sequencer were converted into sequence data (reads) by CASAVA base recognition and clean reads were obtained. Q20, q30, and GC contents of clean reads were calculated (S 1). The transcripts were reconstructed and annotated using StringTie (1.3.3b) [24]. In addition, principal component analysis (PCA) was performed on all samples. DESeq2 [25] software (1.20.0) was used for differential expression analysis between the two comparative combinations. *p*-value ≤ 0.05, FDR ≤ 0.01, and genes with |log2 (foldchange)| > 1 were considered to be significantly differentially expressed genes. In order to predict the main biological and molecular functions of these DEGs, we used gene ontology (GO) classification and functional description. GO includes three aspects: molecular function, cellular components, and biological processes (http://www.geneontology.org/, accessed on 15 June 2020). Rabbit genes were annotated by KEGG (Kyoto Encyclopedia of Genes and Genomes) and the differential gene sets were enriched and analyzed by KEGG pathway. Significance levels for all GO and KEGG terms were corrected by controlling for the false discovery rate (FDR) of multiple pairing comparisons. Statistical software R (R version 4.0.5) and python (Python version 3.9.0) were used for statistical analysis.

## 3. Results

### 3.1. Rabbit Intestinal Tissue Section

**Figure 1:**
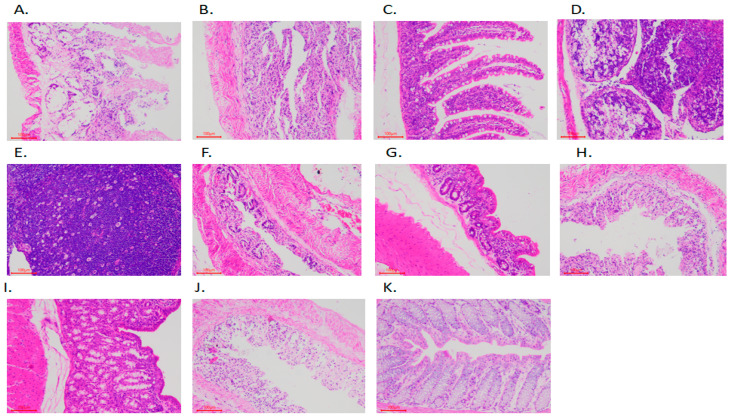
Sections of intestinal tissue samples observed by microscope (HE-staining, 100×). (**A**) The whole layer of coagulative necrosis of the duodenum wall in rabbits with diarrhea. (**B**) The whole layer of coagulative necrosis of the jejunum wall in rabbits with diarrhea. (**C**) The jejunum of healthy rabbits showed normal intestinal structure without obvious histopathological damage. (**D**) The number of lymphocytes in the lymphoid tissue of the lower ileum of diarrheal rabbits decreased seriously, forming a large number of cavities. (**E**) The lymphoid tissue under the ileum of healthy rabbits was rich and arranged in an orderly way. (**F**) The caecum of diarrheal rabbits had coagulative necrosis. (**G**) The intestinal tissue structure of healthy rabbits was normal without obvious histopathological damage. (**H**) The whole layer of coagulative necrosis of the colon wall in rabbits with diarrhea. (**I**) The structure of the colonic intestine in healthy rabbits was normal without obvious histopathological damage. (**J**) The whole layer of coagulative necrosis of the rectum wall in rabbits with diarrhea. (**K**) The structure of the rectum and intestines in healthy rabbits was normal without obvious histopathological damage.

The HE-stained intestinal tissue samples (Figure 1) showed that the intestinal samples of rabbits with diarrhea were mainly manifested in different degrees of necrosis. In severe cases, the whole layer of intestinal wall showed necrosis, and the number of lymphocytes in most intestinal lymph follicles was significantly reduced. In more serious cases, intestinal coagulative necrosis was the main result, followed by mucosal epithelial necrosis and falling off to form erosion, and the clinical manifestation was diarrhea. Conversely, the intestinal structure of rabbits in the CON group was complete and had no pathological characteristics. These results show that feeding an antibiotics-free diet could cause morphological damage to the intestines of rabbits.

### 3.2. Correlation Analysis among Samples and Principal Component Analysis

**Figure 2:**
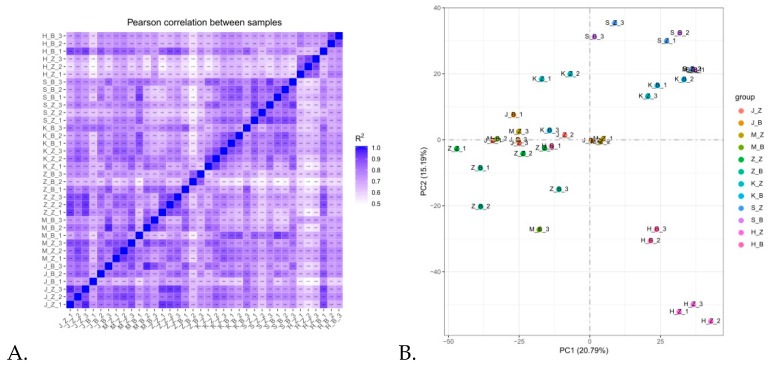
Quantitative analysis of each intestine sample. (**A**) Heat map of correlation between samples. The higher the correlation coefficient between samples, the closer the expression pattern is. (**B**) Principal component analysis result graph. Ideally, the intergroup samples in the PCA diagram should be scattered and the intra-group samples should be clustered together. S_Z: the duodenum of healthy rabbits, S_B: diarrhea in the duodenum of rabbits, H_Z: healthy rabbit ileum, H_B: diarrheal rabbit ileum, K_Z: healthy rabbit jejunum, K_B: rabbits with diarrheal jejunum, M_Z: healthy rabbit cecum, M_B: rabbits with diarrheal cecum, J_Z: healthy rabbit colon, J_B: colon of rabbits with diarrhea, Z_Z: healthy rabbit rectum, Z_B: rectum of rabbits with diarrhea.

The correlation of gene expression level between samples is an important index to test the reliability of the experiment and the rationality of the sample selection. The higher the correlation coefficient between samples, the closer the expression pattern is. It can be observed in Figure 2A that the correlation between gene expression levels among samples of healthy rabbits was generally high (>0.8). Principal component analysis (PCA) was performed for the gene expression values (FPKM) of all samples using the linear algebra method (Figure 2B). It was indicated that the samples within the group were relatively concentrated and the samples between the groups were highly dispersed.

### 3.3. Differential Expression of Genes in Rabbits with Diarrhea

Among all the samples generated from these libraries, rabbits with diarrhea had an average of 45,800,180 double-ended raw reads and 44,413,253 clean reads. Healthy rabbits had an average of 46,213,220 double-ended raw reads and 44,918,133 clean reads. The GC content of the clean readings of all samples was between 44.73–57.14% (Table S1). HISAT2 software was used to quickly and accurately compare clean reads with the reference genome build (orycun2.0 and GCA_ 000003625.1) to obtain the positioning information of reads on the reference genome [26] (Table S2). The Q20 (the reading percentage of phred mass value > 20) was 95.35–98.25% in rabbits with diarrhea and 96.96–97.87% in healthy rabbits. The Q30 of clean reads (the reading percentage of phred mass value > 30) was 88.67–94.99% for rabbits with diarrhea and 92.62–94.03% for healthy rabbits. The mapping rates of all samples of rabbits with diarrhea and healthy rabbits were 81.74–92.84% and 81.94–91.89%, respectively. String Tie software predicted a total of 25,137 new transcripts and 4735 new genes, which were then compared and annotated through go and KEGG databases. Further analysis was conducted based on reference genes.

### 3.4. Functional Enrichment Analysis of Differentially Expressed Genes

**Figure 3:**
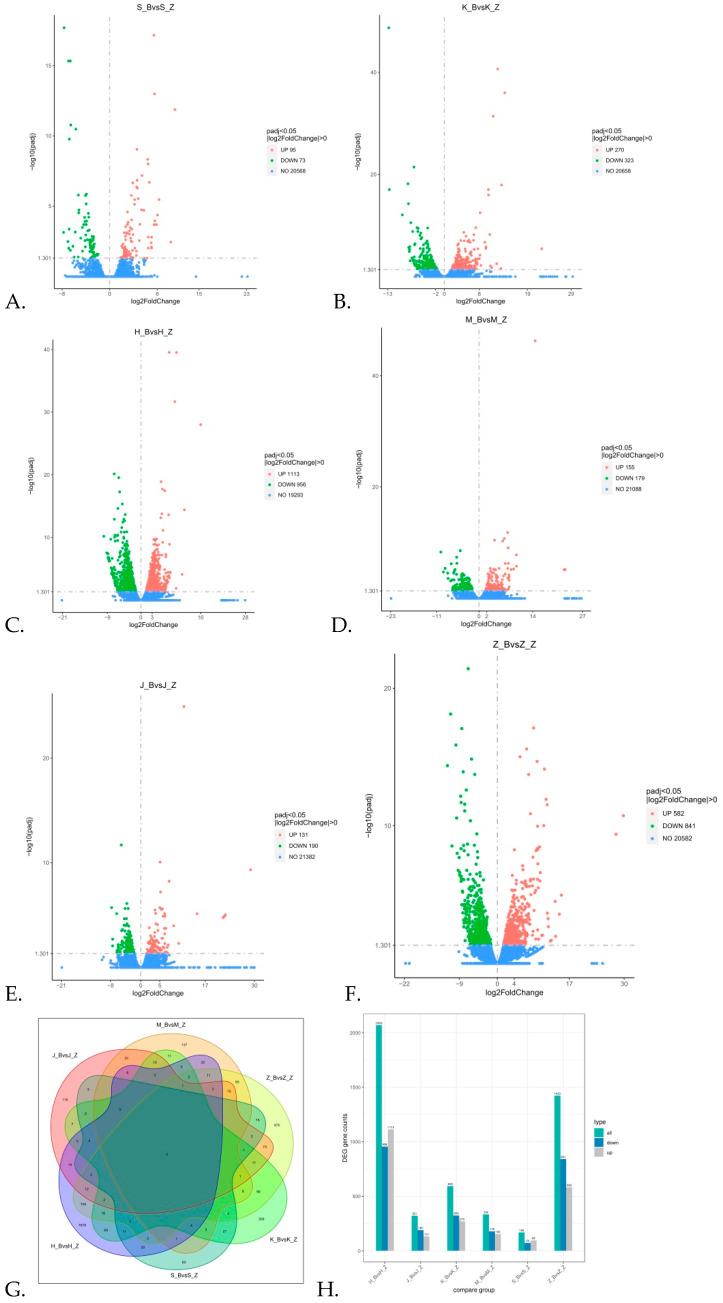
Differential gene volcano map, differential gene Wayne map, and differential gene number histogram. (**A**–**F**) The Vocalo diagram analysis of DEGs in the duodenum, jejunum, ileum, cecum, colon, and rectum in turn. Red indicates significantly upregulated DEG, and blue indicates significantly downregulated DEG (|log2(foldchange)| > 1 and *p*-value ≤ 0.05). (**G**) Differential gene Venn diagram; different colors indicate different comparison combinations. (**H**) The histogram of the number of differentially expressed genes is shown in blue and gray. The number on the column represents the number of differentially expressed genes. S_Z: the duodenum of healthy rabbits, S_B: diarrhea in the duodenum of rabbits, H_Z: healthy rabbit ileum, H_B: diarrheal rabbit ileum, K_Z: healthy rabbit jejunum, K_B: rabbits with diarrheal jejunum, M_Z: healthy cecum of rabbits, M_B: rabbits with diarrheal cecum, J_Z: healthy rabbit colon, J_B: colon of rabbits with diarrhea, Z_Z: healthy rabbit rectum, Z_B: rectum of rabbits with diarrhea.

**Figure 4:**
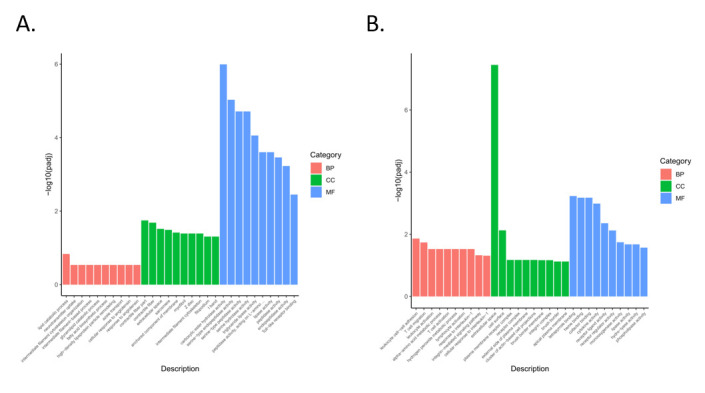

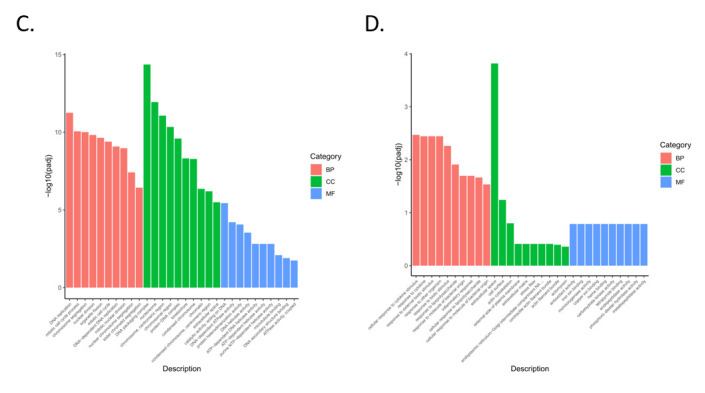

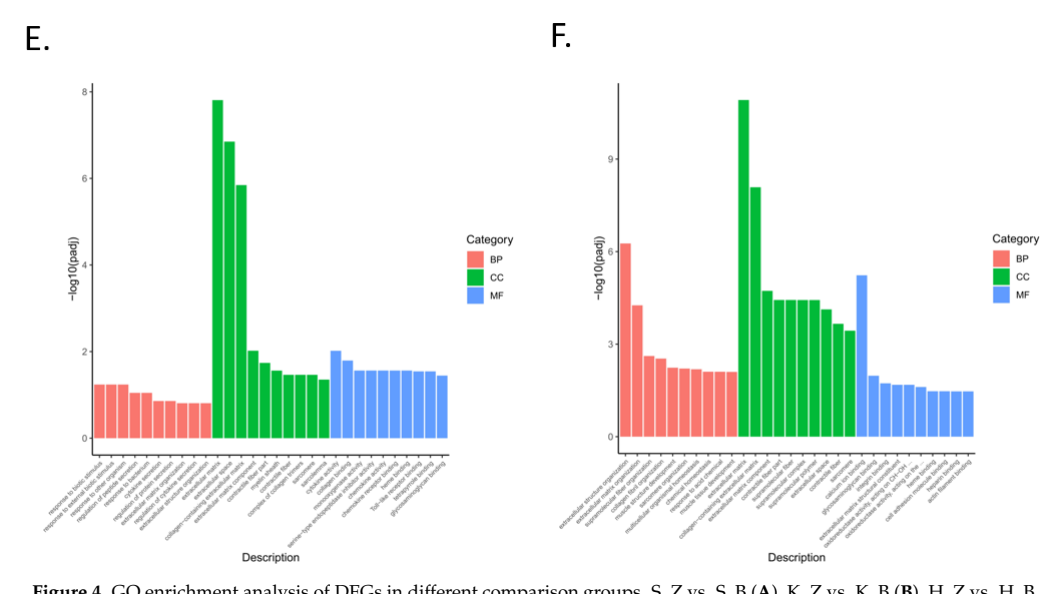
GO enrichment analysis of DEGs in different comparison groups. S_Z vs. S_B (**A**), K_Z vs. K_B (**B**), H_Z vs. H_B (**C**), M_Z vs. M_B (**D**), J_Z vs. J_B (**E**), and Z_Z vs. Z_B (**F**). GO terms are on the *x*-axis. The enrichment ratio of genes is shown as GO terms for BP, CC, and MF. S_Z: the duodenum of healthy rabbits, S_B: diarrhea in the duodenum of rabbits, H_Z: healthy rabbit ileum, H_B: rabbit with diarrheal ileum, K_Z: healthy rabbit jejunum, K_B: rabbits with diarrheal jejunum, M_Z: healthy cecum of rabbits, M_B: rabbits with diarrheal cecum, J_Z: healthy rabbit colon, J_B: colon of rabbits with diarrhea, Z_Z: healthy rabbit rectum, Z_B: rectum of rabbits with diarrhea.

To identify the broad array of genes involved in response to rabbits with diarrhea, we analyzed the genes that responded to the rabbits with diarrhea (Figure 3A–F,H). Comparing DIA and CON, we observed the duodenum, jejunum, ileum, cecum, colon, and rectum in turn. There was a total of 168 DEGs (95 significantly upregulated and 73 significantly downregulated), 593 DEGs (270 significantly upregulated and 323 significantly downregulated), 2069 DEGs (1113 significantly upregulated and 956 significantly downregulated), 334 DEGs (155 significantly upregulated and 179 significantly downregulated), 321 DEGs (131 significantly upregulated and 190 significantly downregulated), and 1423 DEGs (582 significantly upregulated and 841 significantly downregulated), respectively. By analyzing a Venn diagram (Figure 4G), it is evident that there were 63, 329, 1678, 137, 119, and 970 unique differentially expressed genes in the duodenum, jejunum, ileum, cecum, colon, and rectum, respectively.

### 3.5. Enrichment Analysis of GO and KEGG Pathway

Compared with the general description of the properties of genes or transcripts with functional annotation, the lowest level of gene function and KEGG pathway can be annotated by enrichment analyses. In addition, enrichment analysis provides the most detailed information about gene function and KEGG pathway, which can help us to screen unique insights on diarrhea response in rabbits fed a no-antibiotic diet. Furthermore, GO terms and KEGG pathways that satisfy the corrected *p*-value of ≤0.05 were considered significantly enriched. To classify and characterize DEG functions and pathways, we performed a gene ontology (GO) classification and functional annotation of molecular biological function, cellular components, and biological process (Figure 4). Most annotated genes in the biological processes category were related to the inflammatory response and cellular metabolism. DEGs in comparison group S_Z vs. S_B were primarily associated with GO terms related to stimulation, biological processes associated with lipid synthesis, and metabolism, such as the lipid/organic substance catabolic process and the inflammatory/stimulus response. The majority of DEGs in comparison group K_Z vs. K_B were associated with stimulation-related biological processes, G-protein coupled receptor activity, signal transduction-related molecular functions, and cellular components (cytoskeletal part, membrane part, cytoplasm), such as the acute-phase response, G protein-coupled peptide receptor activity, and transmembrane transporter activity. The majority of DEGs in comparison group H_Z vs. H_B were assigned with biological processes associated with stimulus, cellular processes, and cellular components, such as cellular response to endogenous stimulus, negative regulation of biological process, regulation of primary metabolic process, and positive regulation of biological process. The majority of DEGs in comparison group M_Z vs. M_B were associated with stimulus, immune process, cellular processes, signal transduction-related molecular functions, and cellular components, such as response to abiotic stimulus, positive regulation of neutrophil migration, cellular response to interleukin-1, positive regulation of biological process, and intracellular signal transduction. The majority of DEGs in comparison group J_Z vs. J_B were associated with stimulus, immune process, G protein-coupled peptide receptor activity, cellular process, and cellular components, such as cellular response to chemical stimulus, G protein-coupled receptor signaling pathway, cellular response to interleukin-1, neutrophil chemotaxis, positive regulation of cellular process, and cell migration. The majority of DEGs in comparison group Z_Z vs. Z_B were associated with cell development, tissue development, cellular process, and cellular components, such as striated muscle tissue development, striated muscle cell development, muscle structure development, and cell differentiation.

**Figure 5:**
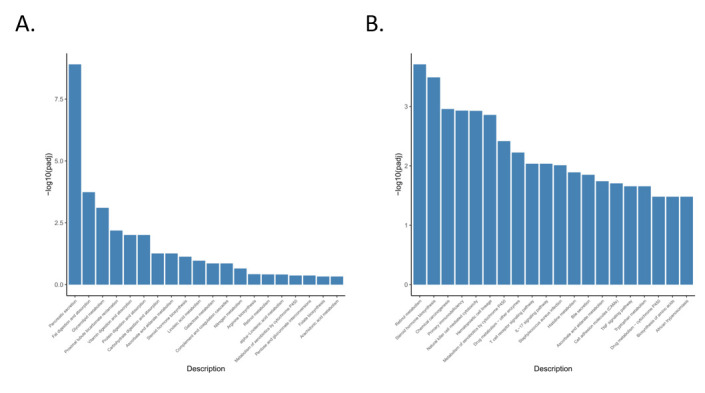

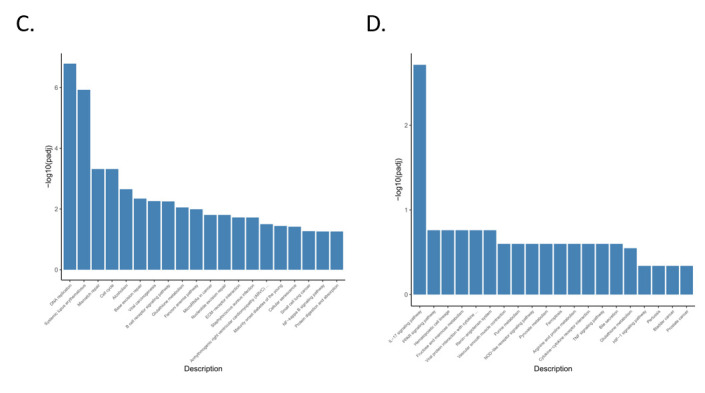

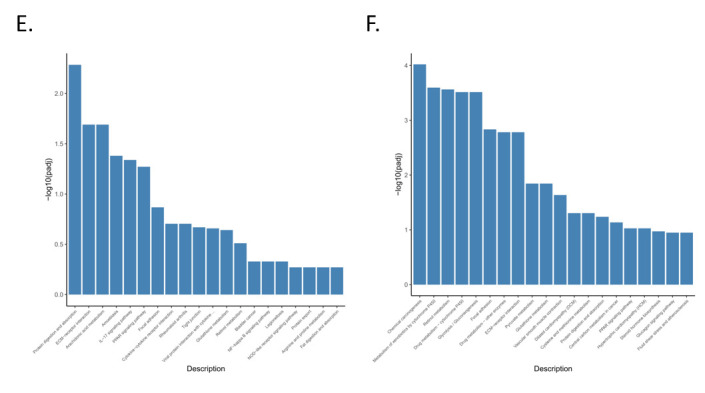
KEGG pathway enrichment analysis of DEGs in different comparison groups. S_Z vs. S_B (**A**), K_Z vs. K_B (**B**), H_Z vs. H_B (**C**), M_Z vs. M_B (**D**), J_Z vs. J_B (**E**), and Z_Z vs. Z_B (**F**). The names of KEGG pathways are on the *x*-axis. S_Z: the duodenum of healthy rabbits, S_B: diarrhea in the duodenum of rabbits, H_Z: healthy rabbit ileum, H_B: diarrheal rabbit ileum, K_Z: healthy rabbit jejunum, K_B: rabbits with diarrheal jejunum, M_Z: healthy cecum of rabbits, M_B: rabbits with diarrheal cecum, J_Z: healthy rabbit colon, J_B: colon of rabbits with diarrhea, Z_Z: healthy rabbit rectum, Z_B: rectum of rabbits with diarrhea.

**Figure 6:**
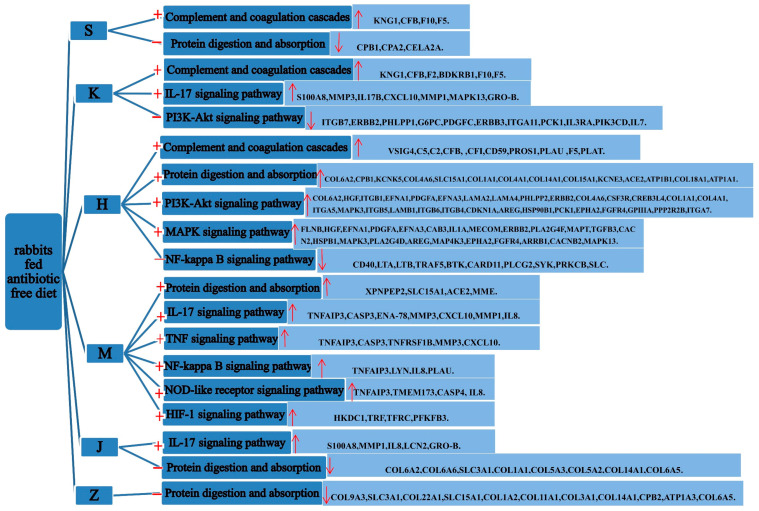
The significantly enriched pathways in DEGs from different intestinal segment comparison groups. S: duodenum; H: ileum; K: jejunum; M: cecum; J: colon; Z: rectum.

Moreover, the analysis identified 9, 21, 25, 5, 12, and 1 KEGG pathways that were significantly enriched in DEGs in comparison groups S_Z vs. S_B, K_Z vs. K_B, H_Z vs. H_B, M_Z vs. M_B, J_Z vs. J_B, and Z_Z vs. Z_B, respectively (Figure 5). The significantly enriched pathways in DEGs from comparison group S_Z vs. S_B mainly included the complement and coagulation cascades and protein digestion and absorption (Figure 6). The pathways significantly enriched in DEGs from comparison group K_Z vs. K_B mainly included complement and coagulation cascades, IL-17 signaling pathway, and PI3K-Akt signaling pathway. The significantly enriched pathways in DEGs from comparison group H_Z vs. H_B mainly included the complement and coagulation cascades, protein digestion and absorption, PI3K-Akt signaling pathway, MAPK signaling pathway, and NF-kappa B signaling pathway. The pathways that were significantly enriched in the DEGs from comparison group M_Z vs. M_B mainly included protein digestion and absorption, IL-17 signaling pathway, TNF signaling pathway, NF-kappa B signaling pathway, NOD-like receptor signaling pathway, and HIF-1 signaling pathway. The pathways that were significantly enriched in the DEGs from comparison group J_Z vs. J_B mainly included protein digestion and absorption, and IL-17 signaling pathway. The pathways that were significantly enriched in the DEGs from comparison group Z_Z vs. Z_B mainly included protein digestion and absorption, chemical carcinogenesis, metabolism of xenobiotics by cytochrome P450, and glycolysis/gluconeogenesis. KEGG pathways that were significantly enriched in more than three comparison groups included NF-kappa B signaling pathway, IL-17 signaling pathway, protein digestion and absorption, NOD-like receptor signaling pathway, metabolism of xenobiotics by cytochrome P450, chemical carcinogenesis, retinol metabolism, and glutathione metabolism. PI3K-Akt, NOD-like receptor, HIF-1, MAPK, and TNF signaling pathways play important roles in immune-related molecular pattern recognition, signal transduction, and host immune system regulation. These results provide important insights into the transcriptional mechanism of intestinal diarrhea induced by a no-antibiotic diet in rabbits.

### 3.6. Gene Expression Levels Are Consistent in Both qRT-PCR and RNA-Seq

**Figure 7:**
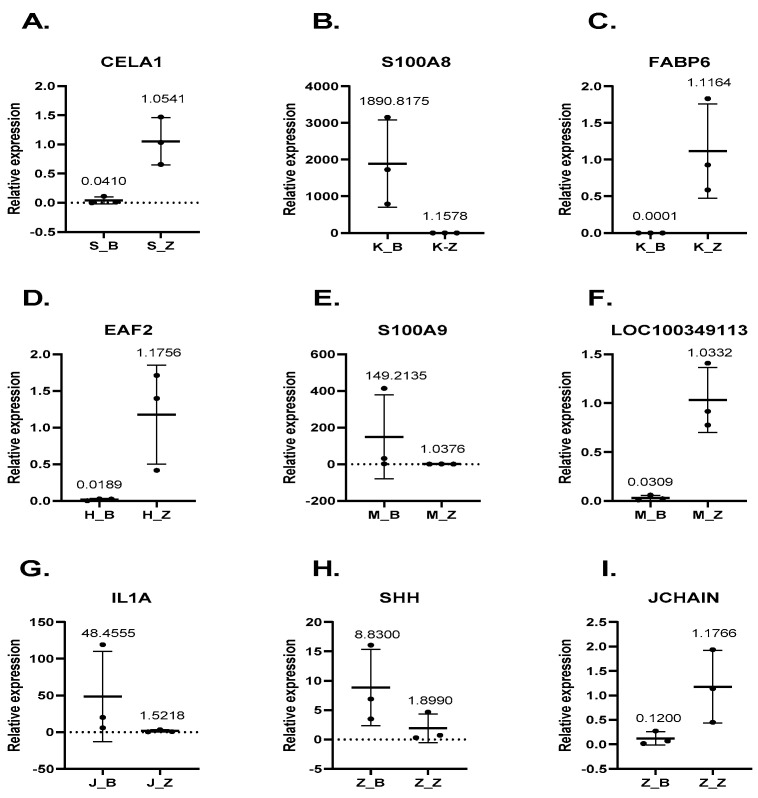
Expression level of genes CELA1, S100A8, FABP6, EAF2, S100A9, LOC100349113, IL1A, CYP4B1, SHH, and JCHAIN validated by RT-qPCR. The β-actin gene was used as an internal control and the relative quantity of gene expression (fold change) of each gene was calculated with the comparative 2^−ΔΔCT^ method. Values (RT-qPCR) shown are mean with SD.

To validate the reproducibility and repeatability of DEGs identified from transcriptome sequencing, we randomly selected 10 genes, namely, CELA1, S100A8, FABP6, EAF2, S100A9, LOC100349113, IL1A, SHH, and JCHAIN, for RT-qPCR analysis (Figure 7). Our results showed that these genes were significantly differentially expressed and were consistently upregulated or downregulated with the gene expression changes based on RNA-seq, which indicated that the RNA-seq data were reliable. 

## 4. Discussion

The promotion and use of an antibiotic-free diet has gradually become an inevitable trend in the development of animal husbandry. However, feeding an antibiotic-free diet would lead to severe diarrhea in rabbits and cause huge economic losses to the rabbit industry. Some studies have shown that diarrhea could be accompanied by intestinal inflammation and intestinal mucosal epithelial necrosis [22,27,28,29,30]. In this experiment, through the observation of intestinal tissue sections, we observed that the intestines of rabbits with diarrhea had different degrees of necrosis, and even intestinal wall coagulative necrosis, mucosal epithelial necrosis, and chylosis. In addition, the number of lymphocytes in most intestinal lymph follicles decreased significantly.

In our study, we used RNA-seq technology to analyze the differential expression profile of the intestinal tract of rabbits with diarrhea fed an antibiotic-free diet for the first time, and verified the accuracy and reliability of transcriptome sequencing data by analyzing the relative expression of the mRNA of various genes. The expression changes of randomly selected genes, which was analyzed by RT-qPCR, were consistently upregulated or downregulated with the gene expression changes based on RNA-seq, and indicated that the RNA-seq data were reliable. Furthermore, we analyzed the functions and pathways of GO and KEGG. For example, KEGG pathway analysis showed that DEGs were significantly enriched in complement and coagulation cascades (ocu04610), protein digestion and absorption (ocu04974), IL-17 signaling pathway (ocu04657), PI3K-Akt signaling pathway (ocu04151), MAPK signaling pathway (ocu04010), NF-kappa B signaling pathway (ocu04064), TNF signaling pathway (ocu04668), NOD-like receptor signaling pathway(ocu04621), HIF-1 signaling pathway(ocu00480), etc. (Figure 6). Compared with CON, we observed that some of the genes involved in the critical process of the intestinal mucosal immune response in rabbits with diarrhea were significantly upregulated or downregulated. In particular, those genes involved in inflammatory response, signal transduction, and immune system-related signal pathways were upregulated.

Complement and coagulation are the main blood-derived proteolytic enzyme cascade reactions that limit bleeding and pathogen invasion [31,32,33], and play a crucial role in innate and adaptive immunity [34]. Notably, the CFB gene is involved in complement cascade regulation and C3 and C5 activation [35,36]. As an important activator, C5 plays an important role in neutrophils, monocytes, and macrophages [37]. Complement also promotes the safe clearance of apoptotic cells and immune complexes, thus helping to alleviate inflammation [38,39]. In this study, the complement and coagulation cascade signaling pathways and DEGs such as KNG1, CFB, F10, F5, F2, C5, and C2 were upregulated in the duodenum, jejunum, and ileum, and may be involved in the process of diarrhea. Moreover, compared with healthy rabbits, diarrhea will directly affect the digestion and absorption of protein. Moreover, studies have shown that intestinal inflammation leads to increased intestinal wall permeability, such as an increase in albumin level in cecum tissue under diarrhea, which may lead to intestinal disease and edema, and may also lead to the obstacles of diarrhea growth compensation, repair, and dysfunction [40]. Genes related to HIF-1 could regulate the transport of ions and water in intestinal mucosal epithelium, thus regulating tissue edema and participating in the inhibitory effect of the intestinal wall on the regulation of transmembrane conductance [41]. Furthermore, studies have shown that the intestine needs abundant hemoglobin to transport oxygen during diarrhea, and the compensatory enhancement of hemoglobin, insufficient blood circulation, and ischemia and hypoxia caused by intestinal mucositis would prevent the self-repair of the intestine, resulting in further serious intestinal damage [42,43]. In this study, the intestinal damage of rabbits with diarrhea caused the decline of immune metabolism ability.

Studies have shown that an imbalance of cytokine secretion could cause intestinal epithelial barrier dysfunction and diarrhea [44]. IL-17 and TNF are inflammatory cytokines associated with diarrhea. IL-17 is secreted by macrophages, dendritic cells (DC), T cells, and other innate cells [45]. In phagocytes, IL-17 participates in inflammatory factor response, establishes a chronic inflammatory state through a self-enhanced positive feedback circuit, and maintains Th17 T-cell population [46,47]. IL-17 and other Th17 cytokines are related to the pathogenesis of a variety of autoimmune and inflammatory responses [48]. In addition, IL-17B and IL-17C stimulates the release of TNF-α and IL-1β in mononuclear cell line THP-1 cells. In this study, IL-17B, IL-8, and several genes related to IL-17 enrichment pathway were significantly upregulated in the jejunum, cecum, and colon of rabbits with diarrhea, such as CXCL10, MMP3, MAPK13, S100A8, GRO-B, MMP1, and IL8. These genes could be used as candidates for important markers of diarrhea-related diseases. Furthermore, pathogen-associated molecular pattern receptors (PAMP) such as Toll-like receptors (TLR) can recognize the pathogen and release a series of related antiviral cytokines. The TLR receptor involved in signal transduction could activate innate immune cells, and express and secrete a variety of proinflammatory cytokines, such as tumor necrosis factor (TNF-α), interleukin (IL), and so on. These cytokines can induce inflammation and promote antigen recognition. In addition, the NOD-like receptor is the cytoplasmic counterpart of TLR, which combines with TLR to form the cytoplasmic membrane and intracellular defense system [49]. In addition, TNF-α regulation may be closely related to the activation of TLR or NOD [50]. The TNF-α enrichment pathway plays a key role in inflammation and produces pleiotropic effects on various cell types [51,52]. TNF-α has significant functional duality and TNF-α can regulate immune function, participate in inflammatory response, release other cytokines, and further stimulate inflammatory response and tissue damage [53,54]. The elevated levels of TNF-α and IL-8 destroy the intestinal mucosal barrier and further deepen diarrhea. In this study, the TNF-α pathway and its related genes, such as TNFAIP3, MMP3, and CXCL10, were significantly upregulated. The NOD-like receptor pathway and its related genes, such as TNFAIP3 and IL8, were significantly upregulated. These DEGs may be important genes involved in intestinal inflammatory response and immune response in rabbits with diarrhea.

It has been reported that activation of the NF-κB pathway in intestinal epithelial cells can cause severe inflammation [55]. NF-κ B is one of the main downstream effectors of TLR activation. It transduces signals together with MAPK and TNF, and TLR/NF in intestinal epithelial cells. The κB signaling pathway is involved in gene transcription of intestinal epithelial cell survival and reconstruction of the intestinal barrier, and therefore serves as an immune mechanism [56]. In addition, the MAPK signaling pathway has anti-inflammatory and anti-apoptotic effects. In this study, the NF-κB signaling pathway in rabbits with diarrhea was upregulated in the cecum, and differential genes such as TNFAIP3 and IL8 were upregulated in the cecum. The MAPK signaling pathway in rabbits with diarrhea was upregulated in the ileum, and differential genes such as MAPK3 and MAPK13 were upregulated in the ileum.

We also analyzed the interactions among the pathways involved in the PI3K-Akt signaling pathway. It is currently considered that the phosphatidylinositol-3-kinase (PI3K) pathway is an important signal transduction pathway for cell-surface receptors to control intracellular activities, and participates in all major leukocyte regulatory activities, such as receptors for antigen recognition and inflammation stimulation [57]. In addition, P13K can regulate the activity of anti-apoptotic signals and inflammatory signals through TNF-α. P13K produces the signaling lipid PtdIns (3,4,5) P3, which activates Akt and affects NF-κB and the response element-binding protein, thereby producing anti-apoptotic signals in granulocytes [58,59,60]. Consistent with previous studies, the PI3K-Akt enrichment pathway in rabbits with diarrhea was downregulated in the jejunum, and differential genes such as IL3RA and IL7 were downregulated. The PI3K-Akt enrichment pathway was upregulated in the ileum and differential genes such as COL6A2, COL4A6, COL1A1, COL4A1, and MAPK3 were upregulated. Therefore, these pathways (Figure 6) and genes (Table S3) should be regarded as potential markers associated with diarrhea.

## 5. Conclusions

In this study, we characterized the transcriptional profiles of rabbits with diarrhea and healthy rabbits after feeding them an antibiotic-free diet. The dynamic changes in differentially expressed genes between rabbits with diarrhea and healthy rabbits are helpful to understand the immune regulation mechanism of rabbits. Our results showed that there were significant differences in the metabolism and the expression of antiviral proteins and genes in the complement system in rabbits fed an antibiotic-free diet. It may be the cause of diarrhea in rabbits. These findings provide a unique insight into the regulatory mechanism of diarrhea in rabbits fed an antibiotic-free diet, and provide some new ideas for future research.

## Data Availability

All data generated or analyzed during this study are included.

## Supplementary Materials

The following are available online at https://www.mdpi.com/article/10.3390/ani11102994/s1, Table S1: Clean reads quality metrics, Table S2: Summary statistics for mapped data of samples, Table S3: Screening of significantly different genes related to diarrhea in rabbits.
