# Supplementary material for: Transcriptomics Analysis Reveals the Immune Response Mechanism of Rabbits with Diarrhea Fed an Antibiotic-Free Diet
Source: Animals (Basel). 2021 Oct 19;11(10):2994. doi: 10.3390/ani11102994 (PMC8532911; doi:10.3390/ani11102994)
Supplement: Supplementary file 1 [file animals-11-02994-s001.zip › animals-1384755-supplementary.pdf]

Table S1. Clean reads quality metrics.

sample	library	raw_reads	clean_reads	clean_bases	error_rate	Q20	Q30	GC_pct
H_B_1	FRAS200021350-1r	45531014	44147252	6.62G	0.03	97.54	93.46	50.41
H_B_2	FRAS200021351-1r	44556194	43117928	6.47G	0.03	97.6	93.41	44.85
H_B_3	FRAS200021352-1r	51102454	49354122	7.4G	0.03	97.4	93.2	47.45
H_Z_1	FRAS200021347-1r	46218568	44740058	6.71G	0.03	97.21	92.85	44.73
H_Z_2	FRAS200021348-1r	41977676	40444974	6.07G	0.03	97	92.71	49.8
H_Z_3	FRAS200021349-1r	45774428	44408618	6.66G	0.03	97.3	93.14	50.86
J_B_1	FRAS200021307-1r	43678660	42569122	6.39G	0.02	98.25	94.99	57.14
J_B_2	FRAS200021310-1r	44315400	42773156	6.42G	0.03	96.78	92.32	48.36
J_B_3	FRAS200021309-1r	45874976	44644908	6.7G	0.03	97.73	93.7	52.61
J_Z_1	FRAS200021304-1r	45824314	44705384	6.71G	0.03	97.75	93.8	50.8
J_Z_2	FRAS200021305-1r	46453394	45365010	6.8G	0.03	97.61	93.54	52.19
J_Z_3	FRAS200021306-1r	43502138	42184332	6.33G	0.03	97.66	93.6	51.27
K_B_1	FRAS200021335-1r	40777484	39636372	5.95G	0.03	96.97	92.56	48.07
K_B_2	FRAS200021336-1r	47977282	46569360	6.99G	0.03	97.1	92.7	45.4
K_B_3	FRAS200021337-1r	45572660	44163540	6.62G	0.03	97.65	93.76	54.08
K_Z_1	FRAS200021332-1r	47465070	46235450	6.94G	0.03	97.63	93.76	54.07
K_Z_2	FRAS200021333-1r	46351422	45191644	6.78G	0.03	97.43	93.31	53.01
K_Z_3	FRAS200021334-1r	45838342	44459204	6.67G	0.03	96.96	92.62	49.73
M_B_1	FRAS200021316-1r	48313798	47079708	7.06G	0.03	97.37	93.12	47.66
M_B_2	FRAS200021319-1r	41740030	40705516	6.11G	0.03	97.93	94.17	52.72
M_B_3	FRAS200021318-1r	45547076	44406510	6.66G	0.03	97.84	93.96	53.55
M_Z_1	FRAS200021313-1r	44674116	43584230	6.54G	0.03	97.59	93.46	48.72
M_Z_2	FRAS200021314-1r	41528492	40285156	6.04G	0.03	97.54	93.46	48.83
M_Z_3	FRAS200021315-1r	47729188	46651768	7.0G	0.03	97.8	93.93	53.01
S_B_1	FRAS200021344-2r	47663190	46724272	7.01G	0.03	95.35	88.67	49.37
S_B_2	FRAS200021342-1r	47012554	45384808	6.81G	0.03	97.6	93.64	48.83
S_B_3	FRAS200021343-1r	46525792	44895756	6.73G	0.03	97.8	94.05	53.69
S_Z_1	FRAS200021339-1r	50940940	49399504	7.41G	0.03	97.54	93.65	52.52
S_Z_2	FRAS200021341-1r	52301622	50286390	7.54G	0.03	97.62	93.45	46.46
S_Z_3	FRAS200021340-1r	46605648	45513426	6.83G	0.03	97.81	94.03	54.12
Z_B_1	FRAS200021329-1r	46152296	44490556	6.67G	0.03	97.56	93.6	53.39
Z_B_2	FRAS200021330-1r	45717174	43829894	6.57G	0.03	97.58	93.71	54.06
Z_B_3	FRAS200021331-1r	46345198	44945774	6.74G	0.03	97.23	92.95	50.86
Z_Z_1	FRAS200021323-1r	43817080	42671884	6.4G	0.03	97.87	94	49.76
Z_Z_2	FRAS200021325-1r	46088580	44917438	6.74G	0.03	97.56	93.49	50.73
Z_Z_3	FRAS200021324-1r	48746950	47481918	7.12G	0.03	97.3	93.24	50.85

NOTE: S_Z: The duodenum of healthy rabbits, S_B: Diarrhea in duodenum of rabbits, H_Z: Healthy rabbit ileum, H_B: Diarrhea rabbit ileum, K_Z: Healthy rabbit jejunum, K_B: Diarrhea rabbit jejunum, M_Z: Healthy cecum of rabbits, M_B: Diarrhea rabbit cecum, J_Z: Healthy rabbit colon, J_B: Colon of diarrhea rabbit, Z_Z: Healthy rabbit rectum, Z_B: Rectum of diarrhea rabbit.

1.sample：sample name

2.library：library number

3.raw_reads：the number of reads in the original data

4.clean_reads：the number of reads after filtering the original data

5.clean_bases：base number of filtered original data (clean base = clean reads * 150bp)

6.error_rate：overall data sequencing error rate

7.Q20：percentage of bases with phred value greater than 20 in total bases

8.Q30：percentage of bases with phred value greater than 30 in total bases

9.GC_pct：Percentage of G and C in four bases of clean reads

Table S2. Summary statistics for mapped data of samples.

sample	total_reads	total_map	read1_map	read2_map
H_B_1	44147252	38371411(86.92%)	17496177(39.63%)	17433187(39.49%)
H_B_2	43117928	40029230(92.84%)	17255892(40.02%)	17213594(39.92%)
H_B_3	49354122	40342646(81.74%)	17464157(35.39%)	17420414(35.3%)
H_Z_1	44740058	40682538(90.93%)	17593789(39.32%)	17551122(39.23%)
H_Z_2	40444974	34331241(84.88%)	15356824(37.97%)	15311976(37.86%)
H_Z_3	44408618	37994723(85.56%)	17303869(38.97%)	17232566(38.8%)
J_B_1	42569122	36204120(85.05%)	14951300(35.12%)	14905066(35.01%)
J_B_2	42773156	36350059(84.98%)	16304042(38.12%)	16191991(37.86%)
J_B_3	44644908	39181226(87.76%)	18478979(41.39%)	18389189(41.19%)
J_Z_1	44705384	39309992(87.93%)	17691412(39.57%)	17628851(39.43%)
J_Z_2	45365010	37578849(82.84%)	17315226(38.17%)	17230099(37.98%)
J_Z_3	42184332	36648853(86.88%)	16407177(38.89%)	16343587(38.74%)
K_B_1	39636372	34218334(86.33%)	15047723(37.96%)	15025446(37.91%)
K_B_2	46569360	41712216(89.57%)	17919103(38.48%)	17860703(38.35%)
K_B_3	44163540	37611429(85.16%)	17289645(39.15%)	17227250(39.01%)
K_Z_1	46235450	39375485(85.16%)	18562969(40.15%)	18577691(40.18%)
K_Z_2	45191644	38493188(85.18%)	18024555(39.88%)	18004705(39.84%)
K_Z_3	44459204	37657826(84.7%)	17015854(38.27%)	17001607(38.24%)
M_B_1	47079708	41829313(88.85%)	18410479(39.1%)	18307040(38.89%)
M_B_2	40705516	36216676(88.97%)	16866159(41.43%)	16822154(41.33%)
M_B_3	44406510	38983466(87.79%)	18355626(41.34%)	18270903(41.14%)
M_Z_1	43584230	38754099(88.92%)	17356320(39.82%)	17293054(39.68%)
M_Z_2	40285156	34846494(86.5%)	15564883(38.64%)	15501707(38.48%)
M_Z_3	46651768	39602947(84.89%)	18602166(39.87%)	18512214(39.68%)
S_B_1	46724272	40681519(87.07%)	17974201(38.47%)	17672922(37.82%)
S_B_2	45384808	39775771(87.64%)	17480172(38.52%)	17443460(38.43%)
S_B_3	44895756	37889057(84.39%)	17707926(39.44%)	17653472(39.32%)
S_Z_1	49399504	40971007(82.94%)	18268898(36.98%)	18198380(36.84%)
S_Z_2	50286390	46205938(91.89%)	19058519(37.9%)	19013192(37.81%)
S_Z_3	45513426	37291551(81.94%)	16803404(36.92%)	16732404(36.76%)
Z_B_1	44490556	38384931(86.28%)	17243448(38.76%)	17269776(38.82%)
Z_B_2	43829894	38083852(86.89%)	18085194(41.26%)	18129669(41.36%)
Z_B_3	44945774	39431910(87.73%)	17948168(39.93%)	17952491(39.94%)
Z_Z_1	42671884	38521594(90.27%)	16460351(38.57%)	16404041(38.44%)
Z_Z_2	44917438	39743164(88.48%)	18262932(40.66%)	18212610(40.55%)
Z_Z_3	47481918	40306833(84.89%)	18328541(38.6%)	18246784(38.43%)

NOTE: S_Z: The duodenum of healthy rabbits, S_B: Diarrhea in duodenum of rabbits, H_Z: Healthy rabbit ileum, H_B: Diarrhea rabbit ileum, K_Z: Healthy rabbit jejunum, K_B: Diarrhea rabbit jejunum, M_Z: Healthy cecum of rabbits, M_B: Diarrhea rabbit cecum, J_Z: Healthy rabbit colon, J_B: Colon of diarrhea rabbit, Z_Z: Healthy rabbit rectum, Z_B: Rectum of diarrhea rabbit.

1. sample: sample name
2. total_ reads: the number of clean reads of sequencing data after quality control
3. total_ map: the number and percentage of reads compared to the genome
4. read1_ map: the number and percentage of read1 compared to the reference genome
5.read2_ map: the number and percentage of read2 compared to the reference genome

Table S3. Screening of significantly different genes related to diarrhea in rabbits.

Tissue	gene_name	gene_id	log2FoldChange	pvalue	Trend
Duodenum	DNASE1	ENSOCUG00000011323	-6.587316965	1.23E-19	↑
	S100A8	ENSOCUG00000009566	7.534476842	3.37E-17	↑
	S100A9	ENSOCUG00000008632	6.395497506	3.37E-12	↑
	KNG1	ENSOCUG00000005003	6.462204636	7.80E-12	↑
	F10	ENSOCUG00000022738	4.984424984	1.45149E-05	↑
	F5	ENSOCUG00000026781	3.106416552	3.82617E-07	↑
	CPB1	ENSOCUG00000003084	-3.169964966	0.0000119	↓
	CPA2	ENSOCUG00000006983	-2.560302791	0.0000289	↓
	CELA2A	ENSOCUG00000008305	-5.661969332	1.74E-14	↓
Jejunum	IL1RN	ENSOCUG00000004161	3.090328443	0.0000216	↑
	IL17B	ENSOCUG00000016145	2.927709689	0.0000286	↑
	S100A8	ENSOCUG00000009566	12.2154977	2.4264E-45	↑
	MMP3	ENSOCUG00000029337	5.938777912	9.91291E-08	↑
	CXCL10	ENSOCUG00000016280	2.338400839	0.000314743	↑
	MMP1	ENSOCUG00000017958	4.534835359	1.84709E-10	↑
	MAPK13	ENSOCUG00000014799	1.903269626	0.000212727	↑
	KNG1	ENSOCUG00000005003	5.261470659	5.58199E-06	↑
	BDKRB1	ENSOCUG00000026468	3.432807704	0.00171423	↑
	F10	ENSOCUG00000022738	9.484662622	9.70959E-09	↑
	F5	ENSOCUG00000026781	2.736123891	0.000417724	↑
	ITGB7	ENSOCUG00000001426	-3.042604213	0.000113156	↓
	ERBB2	ENSOCUG00000010569	-1.723074211	0.000925303	↓
	PHLPP1	ENSOCUG00000015736	-1.858535703	0.00106037	↓
	G6PC	ENSOCUG00000016018	-4.590067528	0.000128402	↓
	PDGFC	ENSOCUG00000017242	-3.101436119	7.89469E-05	↓
	ERBB3	ENSOCUG00000017322	-3.994323867	2.51665E-06	↓
	ITGA11	ENSOCUG00000017726	-2.053676755	0.001166592	↓
	PCK1	ENSOCUG00000017905	-3.904347638	6.48232E-05	↓
	PIK3CD	ENSOCUG00000011054	-3.300429086	0.000760914	↓
	IL15	ENSOCUG00000008827	-2.390981974	0.000348426	↓
	IL7	ENSOCUG00000029611	-2.40562413	0.001151835	↓
	FABP6	ENSOCUG00000005435	-12.72560189	9.85E-54	↓
Ileum	KRT15	ENSOCUG00000013123	3.421254358	4.23E-13	↑
	IL1RN	ENSOCUG00000004161	3.700152565	5.14E-11	↑
	IL1A	ENSOCUG00000008771	4.409391752	2.86E-10	↑
	IL17RC	ENSOCUG00000028217	2.469090726	0.0000075	↑
	IL22RA1	ENSOCUG00000006743	2.496431395	0.004512408	↑
	IL17RD	ENSOCUG00000014051	5.192101269	0.005304381	↑
	CXCL8	ENSOCUG00000011835	4.833400871	9.97E-08	↑
	S100A12	ENSOCUG00000022646	7.57579071	1.5E-44	↑
	S100A8	ENSOCUG00000009566	9.54494115	3.23E-44	↑
	S100A9	ENSOCUG00000008632	9.117676467	3.39E-36	↑
	HGF	ENSOCUG00000001618	4.122210442	0.001390457	↑
	ITGB5	ENSOCUG00000014602	2.735684296	0.0000319	↑
	ITGB4	ENSOCUG00000014951	1.880551732	0.000612556	↑
	ITGB6	ENSOCUG00000014850	2.991003611	0.000665178	↑
	ITGB1	ENSOCUG00000003623	1.885391983	0.001507658	↑
	EFNA1	ENSOCUG00000004307	2.3830876	0.00000753	↑

PDGFA	ENSOCUG00000005660	3.130378862	0.002567281	↑
LAMA2	ENSOCUG00000009427	4.014734373	3.11E-09	↑
LAMA4	ENSOCUG00000009655	1.618041159	0.005787269	↑
PHLPP2	ENSOCUG00000009972	2.933784623	0.001803729	↑
ERBB2	ENSOCUG00000010569	1.876298888	0.002625116	↑
COL4A6	ENSOCUG00000010845	4.530162195	0.000130921	↑
CSF3R	ENSOCUG00000011231	4.645486343	0.005162671	↑
CREB3L4	ENSOCUG00000011784	3.542619894	0.0000023	↑
COL1A1	ENSOCUG00000012881	2.195018335	0.003328664	↑
COL4A1	ENSOCUG00000013232	2.89023724	0.0000443	↑
ITGA5	ENSOCUG00000013369	2.481623679	0.002815669	↑
MAPK3	ENSOCUG00000013716	1.675277073	0.001117228	↑
LAMB1	ENSOCUG00000014676	2.329063172	0.000000352	↑
AREG	ENSOCUG00000016286	7.223454134	0.00000053	↑
HSP90B1	ENSOCUG00000016288	1.894066177	0.000123788	↑
PCK1	ENSOCUG00000017905	2.473914374	0.001042548	↑
EPHA2	ENSOCUG00000000786	2.148686481	0.001806855	↑
FGFR4	ENSOCUG00000003380	2.593117156	0.00000293	↑
PPP2R2B	ENSOCUG00000006861	3.069213977	0.005344859	↑
ITGA7	ENSOCUG00000021735	3.167028823	7.41E-08	↑
VSIG4	ENSOCUG00000004172	3.674652977	0.0000921	↑
C5	ENSOCUG00000005673	6.213569255	0.001313146	↑
C2	ENSOCUG00000006999	3.30612759	1.47E-09	↑
PROS1	ENSOCUG00000025358	2.731983714	0.000802062	↑
PLAU	ENSOCUG00000003155	2.02180218	0.001876809	↑
F5	ENSOCUG00000026781	3.301803353	0.003065155	↑
PLAT	ENSOCUG00000009967	2.800201568	0.00000239	↑
CPB1	ENSOCUG00000003084	6.18811557	0.001405053	↑
SLC15A1	ENSOCUG00000012014	3.940888603	4.22E-09	↑
COL14A1	ENSOCUG00000015234	2.530712514	0.000186874	↑
COL15A1	ENSOCUG00000016236	3.910947665	0.000028	↑
ATP1B1	ENSOCUG00000006867	2.300976561	0.000253737	↑
ATP1A1	ENSOCUG00000001324	2.834557148	0.000000199	↑
FLNB	ENSOCUG00000000828	0.000506469	0.007714336	↑
MECOM	ENSOCUG00000010171	2.721265757	0.002058081	↑
PLA2G4F	ENSOCUG00000011139	1.78861835	0.00127912	↑
MAPT	ENSOCUG00000011386	3.181727217	0.000000836	↑
TGFB3	ENSOCUG00000011415	2.274905311	0.002941936	↑
HSPB1	ENSOCUG00000012690	2.290493822	0.000049	↑
PLA2G4D	ENSOCUG00000013994	6.27751001	0.001715242	↑
MAP4K3	ENSOCUG00000016679	2.081898645	0.003390982	↑
ARRB1	ENSOCUG00000026291	1.748110192	0.000288392	↑
CACNB2	ENSOCUG00000014849	3.044176658	0.0034483	↑
MAPK13	ENSOCUG00000014799	1.428441412	0.003319998	↑
LTBR	ENSOCUG00000001292	2.403394957	0.0000552	↑
CARD10	ENSOCUG00000003517	2.922220459	0.0000056	↑
LTB	ENSOCUG00000006703	-3.447239069	0.000000372	↓
TRAF5	ENSOCUG00000009132	-2.269344546	0.00000817	↓
BTK	ENSOCUG00000010621	-2.979273477	0.00000998	↓
CARD11	ENSOCUG00000011191	-1.82459165	0.001102406	↓
PLCG2	ENSOCUG00000013105	-2.653830763	0.000349697	↓

	SYK	ENSOCUG00000017539	-3.704248675	0.00000117	↓
	PRKCB	ENSOCUG00000013916	-2.698623662	0.0000018	↓
	CXCR5	ENSOCUG00000025048	-3.885431213	0.0000549	↓
	PRKCD	ENSOCUG00000010843	-2.349925607	0.0000403	↓
	EAF2	ENSOCUG00000007081	-5.93489998	9.23E-24	↓
Cecum	S100A9	ENSOCUG00000008632	7.589069605	3.09E-13	↑
	TNFAIP3	ENSOCUG00000002310	2.836096588	0.000230988	↑
	CASP3	ENSOCUG00000006627	2.347740619	8.67585E-06	↑
	MMP1	ENSOCUG00000017958	6.610834208	3.24364E-15	↑
	MMP3	ENSOCUG00000029337	3.155375112	2.32455E-05	↑
	CXCL8	ENSOCUG00000011835	6.106093401	0.00000193	↑
	CXCL5	ENSOCUG00000011838	4.495557183	0.00000368	↑
	CXCL10	ENSOCUG00000016280	3.451294214	0.000298915	↑
	CXCL9	ENSOCUG00000029588	2.51559754	0.003354174	↑
	CXCL11	ENSOCUG00000026515	3.563587747	0.026398696	↑
	IL1A	ENSOCUG00000008771	6.854705898	9.63E-08	↑
	IL22	ENSOCUG00000001663	4.44689955	0.000667969	↑
	IL15	ENSOCUG00000008827	-2.074801258	0.001936108	↑
	IL7R	ENSOCUG00000011402	4.946240741	0.005248886	↑
	IL1RN	ENSOCUG00000004161	2.446741682	0.006173611	↑
	IL36A	ENSOCUG00000014998	4.019428763	0.006314592	↑
	IL13RA1	ENSOCUG00000013687	2.158012914	0.012584525	↑
	IL1B	ENSOCUG00000023958	2.483277924	0.018040664	↑
	IL34	ENSOCUG00000022125	1.852105207	0.021888719	↑
	IL18R1	ENSOCUG00000016476	3.003108488	0.029125813	↑
	TNFRSF1B	ENSOCUG00000014127	2.452962989	0.000995981	↑
	LYN	ENSOCUG00000003056	3.84753878	5.37737E-08	↑
	PLAU	ENSOCUG00000003155	2.251344961	0.000506834	↑
	HKDC1	ENSOCUG00000004938	3.052879221	0.000374782	↑
	TFRC	ENSOCUG00000017033	3.029216799	0.000110669	↑
	PFKFB3	ENSOCUG00000007516	2.403909314	0.000926385	↑
	XPNPEP2	ENSOCUG00000010636	4.884418433	4.92473E-06	↑
	SLC15A1	ENSOCUG00000012014	4.820095128	0.000102679	↑
	MME	ENSOCUG00000017924	4.877219856	3.51715E-05	↑
	DEPP1	ENSOCUG00000006521	-3.859976025	0.000000189	↓
Colon	S100A9	ENSOCUG00000008632	11.40350837	7.16044E-30	↑
	S100A8	ENSOCUG00000009566	6.836516905	0.00051574	↑
	MMP1	ENSOCUG00000017958	6.296652225	7.22121E-09	↑
	IL1A	ENSOCUG00000008771	7.489522523	1.69708E-12	↑
	IL16	ENSOCUG00000010384	-2.46222459	0.000788438	↑
	IL36A	ENSOCUG00000014998	5.593679532	0.000953124	↑
	CXCL8	ENSOCUG00000011835	5.866924546	1.78242E-08	↑
	LCN2	ENSOCUG00000021002	4.229882207	0.000971543	↑
	SLC3A1	ENSOCUG00000005464	-3.999945721	3.27527E-05	↓
	COL14A1	ENSOCUG00000015234	-3.170161016	2.35505E-07	↓
	COL8A1	ENSOCUG00000024465	-3.230690306	4.93783E-06	↓
	COL6A5	ENSOCUG00000024036	-3.767495067	1.40268E-05	↓
	COL6A6	ENSOCUG00000002486	-4.449390866	2.71992E-05	↓
	COL1A1	ENSOCUG00000012881	-2.740241829	8.21544E-05	↓
	COL5A2	ENSOCUG00000015020	-2.403906309	9.26427E-05	↓
	COL5A3	ENSOCUG00000014857	-3.565878254	0.000169806	↓

	CYP4B1	ENSOCUG00000011201	-5.127681597	2.34E-16	↓
Rectum	SHH	ENSOCUG00000016862	8.566049596	1.24E-21	↑
	CXCR2	ENSOCUG00000025638	6.11162353	0.001694478	↑
	IL1A	ENSOCUG00000008771	6.941447969	8.4599E-20	↑
	IL1RN	ENSOCUG00000004161	5.371704372	3.63638E-19	↑
	IL20RA	ENSOCUG00000015696	4.975517876	0.000502545	↑
	IL17RB	ENSOCUG00000013508	-4.316071829	9.07128E-06	↓
	IL7	ENSOCUG00000029611	-2.762003519	6.0368E-05	↓
	IL15	ENSOCUG00000008827	-2.691778359	0.000271998	↓
	SLC15A1	ENSOCUG00000012014	-3.920374009	0.00196346	↓
	SLC3A1	ENSOCUG00000005464	-2.870305682	0.003135879	↓
	COL6A5	ENSOCUG00000024036	-7.857085412	0.000000444	↓
	COL1A2	ENSOCUG00000012264	-3.875026161	0.0000441	↓
	COL3A1	ENSOCUG00000014988	-3.595434196	0.000476194	↓
	COL16A1	ENSOCUG00000005747	-2.721842395	0.001506103	↓
	COL14A1	ENSOCUG00000015234	-2.78679341	0.001766174	↓
	COL11A1	ENSOCUG00000013367	-3.445515279	0.002952154	↓
	CPB2	ENSOCUG00000017940	-4.658268945	0.002255454	↓
	JCHAIN	ENSOCUG00000027219	-6.87932971	2.07E-26	↓

NOTE:

1. Tissue: intestinal tissue
2. gene_name: the name of the gene
3. gene_id: gene number
4. log2FoldChange: The ratio of gene expression level between the treatment group and the control group was processed by the shrinkage model of difference analysis software, and finally the logarithm was taken as the bottom 2
5. pvalue: value of significance test
6. Trend: The gene was significantly up-regulated or down-regulated in the intestine
